# Inhibition and Inactivation of Uropathogenic *Escherichia coli* Biofilms on Urinary Catheters by Sodium Selenite

**DOI:** 10.3390/ijms19061703

**Published:** 2018-06-07

**Authors:** Amoolya Narayanan, Meera S. Nair, Muhammed S. Muyyarikkandy, Mary Anne Amalaradjou

**Affiliations:** 1 Department of Psychology, University of Connecticut, Storrs, CT 06269, USA; amoolya.narayanan@gmail.com; 2 Department of Animal Science, University of Connecticut, Storrs, CT 06269, USA; meera.nair@gmail.com (M.S.N.); muhammed.muyyarikkandy@uconn.edu (M.S.M.)

**Keywords:** UPEC, selenium, biofilm, urinary catheters, exopolysaccharide, gene expression

## Abstract

Urinary tract infections (UTI) are the most common hospital-acquired infections in humans and are caused primarily by uropathogenic *Escherichia coli* (UPEC). Indwelling urinary catheters become encrusted with UPEC biofilms that are resistant to common antibiotics, resulting in chronic infections. Therefore, it is important to control UPEC biofilms on catheters to reduce the risk for UTIs. This study investigated the efficacy of selenium for inhibiting and inactivating UPEC biofilms on urinary catheters. Urinary catheters were inoculated with UPEC and treated with 0 and 35 mM selenium at 37 °C for 5 days for the biofilm inhibition assay. In addition, catheters with preformed UPEC biofilms were treated with 0, 45, 60, and 85 mM selenium and incubated at 37 °C. Biofilm-associated UPEC counts on catheters were enumerated on days 0, 1, 3, and 5 of incubation. Additionally, the effect of selenium on exopolysacchride (EPS) production and expression of UPEC biofilm-associated genes was evaluated. Selenium at 35 mM concentration was effective in preventing UPEC biofilm formation on catheters compared to controls (*p* < 0.05). Further, this inhibitory effect was associated with a reduction in EPS production and UPEC gene expression. Moreover, at higher concentrations, selenium was effective in inactivating preformed UPEC biofilms on catheters as early as day 3 of incubation. Results suggest that selenium could be potentially used in the control of UPEC biofilms on urinary catheters.

## 1. Introduction

Urinary tract infection (UTI) is one of the most common infections in humans, accounting for more than 150 million cases worldwide [1]. In the U.S., approximately 11 million cases of UTIs are reported each year with associated costs estimated to be $5 billion annually [2]. In addition to being the most common bacterial infection, UTIs also account for 36% of all health-care associated infections (HAI; [3]). Of these 36% infections, 80% of them are estimated to be catheter-associated [3,4]. In fact, catheter-associated urinary tract infections (CAUTIs) are the most common HAI in the United States, accounting for 1 million cases each year [5,6]. According to the Centers for Disease Control and Prevention (CDC), a CAUTI is defined as a UTI in a patient who had an indwelling urinary catheter in place at the time or within 48 h prior to infection [7]. Beyond the initial urinary infection, CAUTIs can lead to complications including bacteremia, endocarditis, osteomyelitis, septic arthritis, and meningitis [4]. These pathologies collectively result in prolonged hospital stays and increased morbidity and mortality [8,9].

The susceptibility of an individual to CAUTI is mediated by several risk factors, including older age, female gender, diabetes, and impaired immunity [9,10]. However, the most important risk factor is the use of an indwelling catheter and the duration of catheterization [9,11]. The CDC estimates that approximately 12–16% of adult hospital inpatients will have an indwelling catheter at some point during their hospitalization and that the risk for developing a CAUTI increases by 3–7% with each additional day of catheterization [7]. This increased susceptibility in catheterized patients is due to the ability of the catheter to bypass several host defenses and thereby enable bacterial entry into the urinary tract [12]. In intubated patients, bacteria can ascend from the urethral meatus into the bladder by migrating between the mucosal and catheter surfaces. Further, contamination of the drainage bag or disruption in the tubing junction may also result in bacterial migration via the drainage system [4]. Additionally, the presence of an indwelling device favors the persistence of the etiological agent in the urinary tract, thereby increasing the risk for CAUTI [13].

UTIs, including CAUTIs, can be caused by several bacterial species, including uropathogenic *Escherichia coli* (UPEC), *Staphylococcus saprophyticus*, *Klebsiella pneumoniae*, *Enterococcus faecalis*, group B *Streptococcus*, *Staphylococcus aureus*, *Proteus mirabilis*, *Pseudomonas aeruginosa*, and *Enterobacter* spp. [9,14]. However, the most common causative agent for UTI and CAUTI is UPEC. It is estimated that UPEC is responsible for approximately 80% of all UTIs [9,14]. This high incidence of infections associated with UPEC can be attributed to the repertoire of virulence factors that each UPEC strain possesses [1,4]. These factors, which are critical to establishing a successful infection, include adhesins, toxins, surface polysaccharides, flagella, iron acquisition systems, immune evasion, and biofilm formation [1,9]. In case of CAUTI, the ability of the pathogen to adhere and form biofilms on the indwelling medical device results in persistent and recurrent infections in catheterized patients [4,15,16].

In order to form a biofilm, the pathogen must first adhere to the medical device long enough to facilitate permanent attachment. In case of catheters, this adhesion and attachment is favored by the deposition of proteins and polysaccharides from the urine on to the device surface. Once the bacteria are permanently attached to the catheter surface, they produce exopolysaccharides to from the biofilm. Several studies have demonstrated the presence of adherent biofilms on catheters removed from patients with CAUTI [17,18,19]. Besides providing them with the ability to persist in an environment, once encased in a biofilm, pathogens are protected from environmental conditions, antibiotic therapies, and the host’s immune system [15,20]. As with other bacterial infections, antibiotics are the current therapeutic agents of choice in controlling CAUTIs. However, given the inherent resistance of a biofilm to antimicrobial agents and the increase in rates of antibiotic resistance, alternative therapies are warranted [9]. Therefore, the present study investigated the potential application of a dietary mineral, namely selenium, in controlling UPEC biofilms on catheters.

Selenium (Se) is a well-recognized dietary antioxidant, and its compounds are commonly used in nutrition and chemoprevention therapy [21]. It is an essential component of several enzymes, including glutathione reductase, and is recommended for daily dietary intake in humans by the Food and Drug Administration (FDA) with an upper tolerable intake level of 400–800 µg [22]. Several selenium-based formulations, such as selenomethionine and sodium selenite (Na_2_SeO_3_), are available commercially as food supplements, anticancer agents, and immune stimulators. Although widely known for its antioxidant properties, selenium as an antibacterial agent is a novel compound that has not been widely explored [23]. There are a few studies that have investigated the antibacterial potential of selenium and its compounds. Vasic et al. [24] demonstrated the ability of sodium selenite to alter the growth dynamics and extracellular protein synthesis in *Bacillus subtilis*, *Bacillus mycoides*, *E. coli*, and *Pseudomonas* spp. The study also showed that sodium selenite increased the inhibitory effect of antibiotics for all tested bacteria in liquid culture. Similarly, Kumar et al. [25] observed an anti-ulcer and antibacterial activity in rats infected with Helicobacter pylori. Further, Alam et al. [26] demonstrated that sodium selenite exhibited a strong spectrum of activity against planktonic cells of *Bacillus subtilis*, *Staphylococcus aureus*, *Escherichia coli*, and *Klebsiella planticola*. Furthermore, biosynthesized Se nanoparticles were found to be inhibitory to foodborne pathogens, including *Bacillus cereus*, *Enterococcus faecalis*, *S. aureus*, *E. coli* O157:H7, *S. typhimurium*, and *S. enteritidis* [27,28]. Although these studies demonstrate the antibacterial activity of selenium, to the best of our knowledge and based on a survey of current literature, there is no report on the antibiofilm effect of sodium selenite. Consequently, this study investigated the efficacy of sodium selenite in inhibiting UPEC biofilm formation and inactivating mature biofilms on urinary catheters. Further, the effect of selenium on the expression of virulence genes essential for UPEC adhesion and biofilm formation was studied.

## 2. Results and Discussion

The sub inhibitory concentration (SIC), minimum inhibitory concentration (MIC), and MBC minimum bactericidal concentration (MBC) of selenium against UPEC was 35 mM (0.6%), 60 mM (1%), and 85 mM (1.5%), respectively. Since selenium was found to be equally effective in inhibiting and inactivating UPEC biofilms in both tested strains (*p* > 0.05), only results observed with *E. coli* CFT073 are presented in the manuscript unless mentioned otherwise.

### 2.1. Inhibition of UPEC Biofilm Formation on Urinary Catheters by Selenium

Since the initial adhesion, attachment, and exopolysaccharide (EPS) production are critical to the establishment of a mature biofilm, compounds that inhibit the growth of bacteria have been routinely used to impair biofilm formation [29,30,31,32,33]. However, since these compounds may lead to selection for strains resistant to the antimicrobial, there has been an increasing interest in the use of agents that regulate biofilm formations without interfering with bacterial growth. Therefore, in the present study we investigated the ability of an SIC of selenium to inhibit biofilm formation as means to reduce the risk for biofilm-associated pathologies, including CAUTI. Since selenium is employed at the SIC, it is expected that the antimicrobial metal would inhibit biofilm formation by regulating the process and not by inactivating the organism.

**Figure 1:**
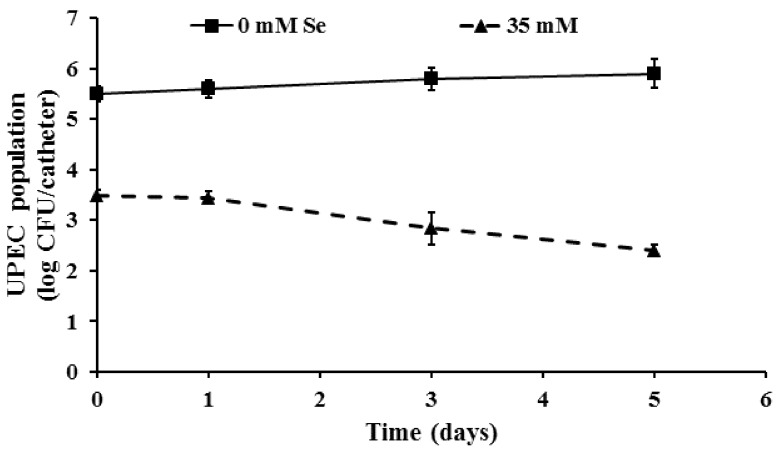
Inhibition of uropathogenic *Escherichia coli* (UPEC) biofilm formation on urinary catheters by selenium. Catheter pieces (3 cm) were inoculated with UPEC (~6.0 log CFU) and incubated at 37 °C in the absence and presence of a sub inhibitory concentration (SIC) of selenium for 5 days. Following 0, 1, 3, and 5 days of exposure, the surviving cells in the biofilm were dispersed by sonication and enumerated. Data are represented as mean ±SEM. Treatments are significantly different from the control at *p* ≤ 0.05.

As can be seen from Figure 1, incorporation of the SIC of selenium significantly inhibited biofilm formation when compared to the control (*p* ≤ 0.05). With the control samples (0 mM Se), on day 1, approximately 5.5 log CFU of biofilm-associated UPEC were observed. On the other hand, in the presence of 35 mM selenium, UPEC counts in the biofilm were reduced to ~3.45 log CFU/catheter, respectively. Further, by the end of the 5-day incubation, selenium treatments reduced the number of biofilm-associated bacteria by 3.5 log when compared to the control (~6.0 log CFU/catheter). Similar results were observed when *E. coli* O157:H7 and *Vibrio cholera* were exposed to the SIC of selenium, manganese, and zinc [34,35]. Specifically, at 24 °C, the use of 30 mM selenium was found to reduce the number of biofilm-associated *E. coli* O157 population by approximately 4 log CFU/mL when compared to the control (~8 log CFU/m; [34]). Along these lines, Yang et al. [36] demonstrated that sub-MIC of Allicin, an essential oil in garlic, inhibited UPEC biofilm formation on a polystyrene matrix.

### 2.2. Effect of Selenium on EPS Production in UPEC Biofilms

Bacterial cells within the biofilm are encased in an extracellular matrix (ECM) that helps to tenaciously bind the sessile cells to the underlying surface [32]. The ECM is highly hydrated and primarily composed of exopolysaccharides [4,32]. In addition to EPS, the matrix is also composed of proteins, DNA, lipids, glycolipids, and membrane vesicles [5,15,37,38]. Besides anchoring the biofilm to the underlying substrate, ECM is also essential to the integrity and function of the biofilm. Specifically, any defect in EPS synthesis and export can impair the formation of multilayer biofilms in most bacteria [39,40,41].

**Figure 2:**
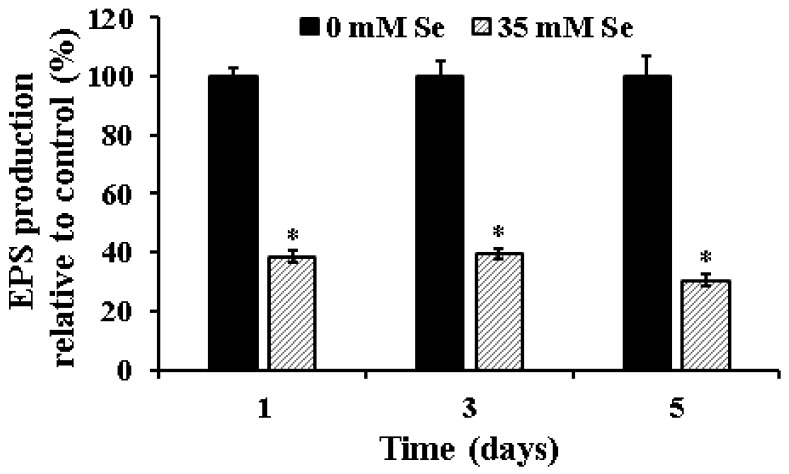
Effect of selenium on exopolysaccharide (EPS) production by UPEC. Sterile 96-well polystyrene tissue culture plates were inoculated with 200 µL of the cell suspension (~6.0 log CFU) followed by the addition of 0 or 35 mM selenium. The plates were incubated at 37 °C. Following 1, 3, and 5 days of exposure, EPS production was assayed by staining the wells with 0.01% ruthenium red and recording the absorbance at 450 nm. Data are represented as mean ± SEM. * Treatments are significantly different from the control at *p* ≤ 0.05.

Given the significance of EPS to the structural integrity of the biofilm and our previous observation of the inhibitory effect of selenium on UPEC biofilm formation, we investigated the efficacy of the SIC of selenium to inhibit EPS production and thereby impair biofilm formation. As can be seen in Figure 2, exposure to the SIC of selenium significantly impaired EPS production by UPEC (*p* ≤ 0.05). As early as day 1 of incubation, EPS production in the treatment was found to be reduced by greater than 60% when compared to the control. Further, the reduced ability to produce EPS was observed till the end of the 5-day study period with the selenium-treated samples. Additionally, this reduction in EPS production was concomitantly associated with a reduction in the number of biofilm-associated UPEC (Figure 1). On the other hand, with the control samples, there was an overall increase in EPS production with the maturation of the biofilm from day 1 to day 5. Our results are in corroboration with previous findings that demonstrate that exposure of *E. coli*, *K. pneumoniae*, *E. faecalis*, *S. aureus*, and *B. subtilis* to turmeric extract resulted in a significant reduction in EPS production and associated biofilm formation [42]. Similarly, inclusion of 1% ginger extract was found to significantly reduce EPS production and biofilm formation by 40–69% in *P. aeruginosa* PA14 [33]. Moreover, use of selenium (30 mM; SIC) has been recently shown to significantly reduce EPS production at 24 °C in *E. coli* O157 by greater than 75% by 96 h of incubation [34].

### 2.3. Effect of Selenium on the Expression of Biofilm-Associated Genes in UPEC

As in other bacteria, the transition of planktonic UPEC to a sessile, sedentary biofilm requires a precisely orchestrated and coordinated regulation of genes critical to biofilm formation [43]. Since incorporation of selenium at its SIC led to a significant reduction in UPEC biofilm formation in the absence of a bacteriostatic or bacteriocidal effect, we performed RT-qPCR on UPEC genes (*fimA*, *fimH*, *papG*, *focA*, *sfaS*) associated with biofilm formation and virulence to understand the underlying mechanism of action. These gene candidates were selected based on previous studies demonstrating their role in UPEC biofilm formation and virulence. For instance, in UPEC, the initial adhesion that is required to trigger biofilm formation is mediated by the Type I pilli [4,36,44]. The two major units of the type I pili are FimH and FimA encoded by the *fimH* and *fimA* genes, respectively [45,46]. With specific reference to biofilm formation, numerous studies have demonstrated the role of the FimH adhesion in mediating UPEC attachment to biotic and abiotic surfaces [47,48,49]. Further, UPEC *fimH* deletion mutants were found to be severely defective in pellicle formation when compared to the wild-type strain [50]. Similarly, *fimA* has been shown to be involved in the attachment of Gram-negative bacteria, including *E. coli*, to abiotic surfaces [51]. Towards this, Silva et al. [52] observed significant upregulation of *fimA* gene expression in *E. coli* during biofilm formation when compared to planktonic cells. In corroboration with these studies, a 2–3 fold reduction in *fimH* and *fimA* expression was observed with selenium-treated UPEC when compared to the control. Therefore, the ability of selenium to inhibit biofilm formation could be attributed to the impaired initial adhesion process.

**Figure 3:**
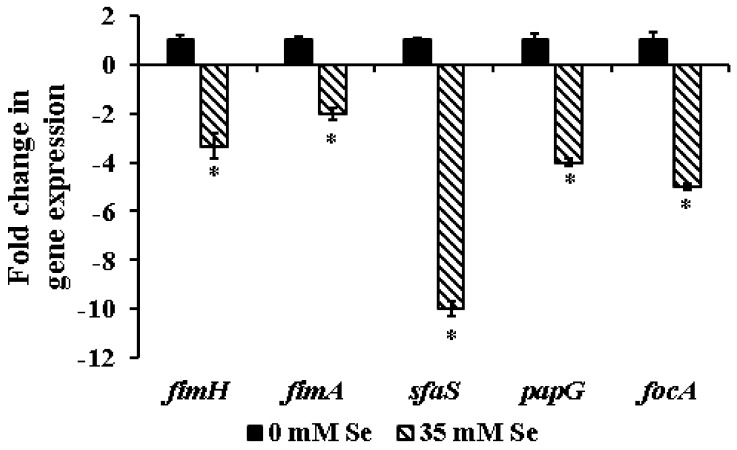
Effect of sub-inhibitory concentrations of selenium on the expression of biofilm genes in UPEC. Relative gene expression was assayed using the StepOne Plus Real Time PCR System. The data were normalized to the endogenous control and the level of candidate gene expression between selenium-treated and untreated samples was compared to study relative gene expression. * Treatments are significantly different from the control at *p* ≤ 0.05.

Similarly, a 4–10 fold reduction in *focA*, *sfaS*, and *papG* expression was also observed following selenium treatment of UPEC (Figure 3). These genes encoding for the type I pili, P (*pap*), F1C (*focA*), and S-fimbriae (*sfaS*) are considered to be important virulence factors for the induction of UTI and formation of biofilm [53,54,55,56]. A higher prevalence of *sfaS* (S-fimbriae) gene was observed for UPEC strains producing moderate to strong biofilms [57]. Similarly, Adamus-Bialek et al. [55] and Naves et al. [58] demonstrated that the virulence genes *sfa*, *foc*, and *papG* were more common in strong biofilm-producing *E. coli* isolates. Hence, in addition to reducing EPS production, the inhibitory effect of selenium on UPEC biofilm formation could also be mediated by the downregulation of the above-mentioned genes. Also, it is important to note that these adhesins play a critical role in UPEC attachment, invasion, and colonization in the urinary tract [59]. Hence, besides inhibiting biofilm formation, selenium at the SIC level could also serve as an anti-virulence agent in attenuating the infectious nature of UPEC.

### 2.4. Inactivation of Pre-Formed UPEC Biofilms on Urinary Catheters by Selenium

Microbial biofilms are of significant public health concern given their reduced susceptibility to antimicrobial agents and their ability to cause infections in patients with indwelling medical devices [16,32,60]. The increased resistance to antimicrobials of biofilms can be attributed to the extensive ECM, reduced growth rates of biofilm-associated bacteria, and the protective environment within the biofilm [61]. The presence of ECM provides a significant hurdle to the diffusion of the antimicrobial into the biofilm, thereby limiting its contact and antimicrobial effect on the bacteria within the biofilm. Further, studies have shown that the EPS of *P. aeruginosa* can bind to tobramycin and limit the entry of the antibiotic into the biofilm, thus reducing its antimicrobial susceptibility [62]. Similar studies have demonstrated that antimicrobial metals, such as zinc and manganese, exhibit a reduced antibiofilm efficacy due to their decreased diffusion through the biofilm [63,64]. Hence, in the present study, higher concentrations of selenium (MIC and MBC) were employed in the biofilm inactivation studies.

**Figure 4:**
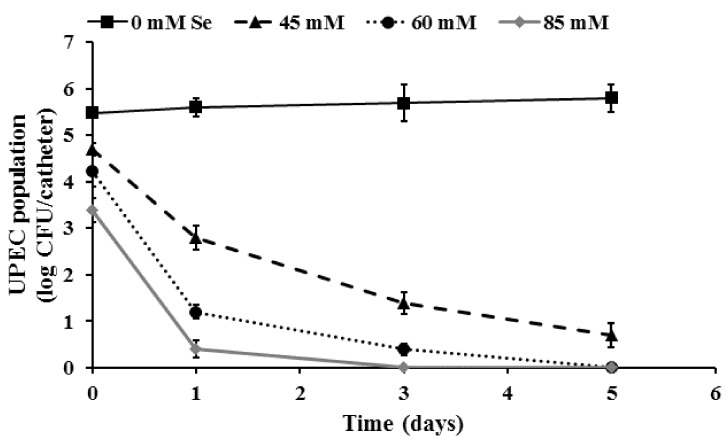
Inactivation of UPEC biofilm on urinary catheters by selenium. Catheter pieces (3 cm) were inoculated with UPEC (~6.0 log CFU) and incubated at 37 °C for biofilm formation. The established biofilm was treated with 0, 45, 60, and 85 mM of selenium for an exposure time of 0, 1, 3, and 5 days. Following exposure, the surviving cells in the biofilm were dispersed by sonication and enumerated. Data are represented as mean ± SEM. Treatments are significantly different from the control at *p* ≤ 0.05.

**Figure 5:**
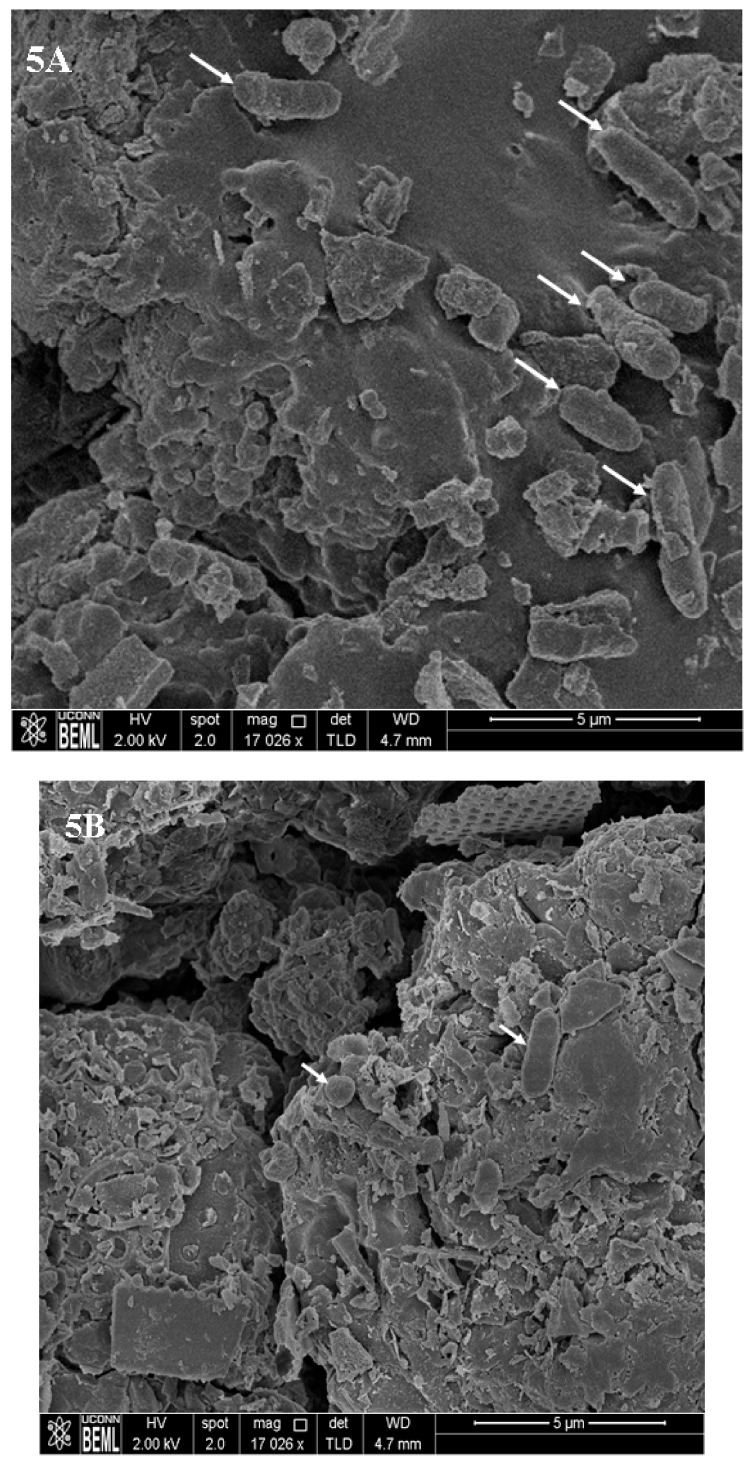
Ultrastructure images of UPEC biofilm on urinary catheters. Catheter pieces (3 cm) were inoculated with UPEC (~6.0 log CFU) and incubated at 37 °C for biofilm formation. The established biofilm was treated with 0 and 85 mM of selenium for an exposure time of 1 day. Following exposure, the catheter pieces were processed for SEM imaging. (**A**) Inoculated catheter without treatment, (**B**) inoculated catheter after selenium treatment. Arrows indicate UPEC cells on the catheter surface.

As can be seen from Figure 4, treatment of preformed biofilms with selenium (45–85 mM) was effective in significantly killing fully formed UPEC biofilms on urinary catheters (*p* < 0.05). With the untreated control samples, approximately 5.5 and 5.9 log CFU of UPEC was recovered from the catheter biofilms on day 1 and day 5, respectively. However, with selenium treatment, a dose-dependent antibiofilm effect was observed until the end of the study. Of the three concentrations tested, 85 mM selenium (MBC) was found to be most effective with a greater than 5 log (92%) reduction and complete inactivation of the UPEC biofilm on day 1 and day 3 of the study, respectively. Similarly, treatment with 60 mM (MIC) and 45 mM (sub-MIC) lead to an ~5 log reduction in biofilm populations by day 3 and day 5, respectively. In addition to bacterial enumeration, SEM was performed to visualize the architecture and morphology of UPEC biofilms formed on urinary catheters. The SEM image of UPEC biofilm grown on urinary catheters is depicted in Figure 5A. These images confirm the presence of biofilm growth on the catheter pieces, where individual UPEC cells can be seen scattered over the matrix surface (Figure 5A). In addition, analysis of the cross-section and inner surface of the catheter samples revealed that the relative number of bacteria on the catheter pieces treated with selenium (Figure 5B) was significantly less compared to the untreated samples (Figure 5A).

## 3. Materials and Methods

### 3.1. Bacterial Isolates and Growth Conditions

Two UPEC strains (*E. coli* CFT073 and *E. coli* UMN 026) obtained from BEI resources (Manassas, VA, USA) were used in the study. All bacteriological media used in the study were procured from Difco (Becton, Dickson and Company, Franklin Lakes, NJ, USA). Each UPEC strain was cultured separately in Tryptic Soy broth (TSB) at 37 °C overnight. After incubation, the cultures were centrifuged (3000× *g*, 12 min, 4 °C), and washed twice in phosphate-buffered saline (PBS, pH 7.0), separately. The pellet was then resuspended in PBS and used as the inoculum. Bacterial counts in the UPEC cultures were confirmed following serial dilution and plating on Tryptic Soy agar (TSA) plates followed by incubation at 37 °C for 24 h.

### 3.2. Determining the SIC, MIC, and MBC of Se against EHEC

The SIC (sub-inhibitory concentration), MIC (minimum inhibitory concentration), and MBC (minimum bactericidal concentration) of Se against UPEC were determined as previously described [59]. Briefly, sterile 24-well polystyrene tissue culture plates (Costar, Corning Incorporated, Corning, NY, USA) containing TSB (1 mL/well) were inoculated separately with ~5.5 log CFU of UPEC followed by the addition of 0 to 5 µL of 50% stock solutions of Se (sodium selenite, Sigma-Aldrich, St. Louis, MO, USA) with an increment of 0.5 µL. The plates were incubated at 37 °C for 24 h, and bacterial growth was determined by culturing on TSA plates. The highest concentration of Se that did not inhibit bacterial growth after 24 h of incubation when compared to the control samples was selected as the SIC for this study. The lowest concentration of Se that inhibited bacterial growth after incubation was taken as the MIC of the treatment and the lowest concentration of Se that reduced UPEC population by ~5.0 log CFU/mL after incubation at 37 °C for 24 h was taken as the MBC.

### 3.3. Inhibition of UPEC Biofilm Formation on Urinary Catheters by Selenium 

The efficacy of selenium to inhibit UPEC biofilm formation on urinary catheters was determined according to a previously published protocol [20,65]. Latex 12Fr Foley urinary catheter (AtHomeMedical, Morris Plains, NJ, USA) was cut into 3-cm pieces. Each catheter piece was sealed at one end, filled with 1 mL of bacterial culture (~5.5 log CFU), and sealed at the other end. The catheter pieces were then incubated at 37 °C for 30 min to facilitate UPEC attachment to the catheter luminal surface. Following incubation, the catheter pieces were washed with sterile PBS to remove unattached cells and transferred to sterile tubes containing 10 mL TSB with 0% (control) or 0.6% (35 mM) selenium. The catheter pieces were then incubated statically at 37 °C for 5 days to facilitate biofilm formation. On days 0, 1, 3, and 5, the biofilm-associated UPEC population was determined after dislodging the biofilm from the luminal surface. This was achieved by vortexing the catheter pieces in separate tubes containing 10 mL of PBS for 1 min followed by sonication at 40 KHz for 5 min in a bath sonicator (Branson, North Olmstead, OH, USA). After sonication, viable bacterial counts in PBS from each tube were enumerated after serial dilution (1:10 in PBS) and plating on duplicate TSA plates [20,66]. Three catheter pieces were included per treatment at each sampling time and the experiment was repeated three times.

### 3.4. Effect of Se on UPEC Exopolysaccharide (EPS) Production

The efficacy of selenium in reducing EPS production in UPEC biofilms was determined by ruthenium red staining assay [67]. For the assay, sterile 96-well polystyrene tissue culture plates (Costar) were inoculated with 200 µL (~5.5 log CFU) of overnight, PBS-washed UPEC cultures followed by the addition of the SIC of selenium. The plates were incubated at 37 °C for 5 days. After 1, 3, and 5 days of incubation, the biofilms developed were scraped and washed with PBS followed by the addition of 200 μL of aqueous 0.01% ruthenium red (Sigma-Aldrich) to each well and incubation at 25 °C for 60 min. The optical density (450 nm) of the residual stain was then measured using a synergy HT multi-mode microplate reader (BioTek, Winooski, VT, USA). The amount of dye bound to the biofilm was determined by the formula OD_BF_ = OD_B_ − OD_S_ (OD_BF_: optical density (450 nm) of biofilms; OD_B_: optical density (450 nm) of blanks; OD_S_: optical density (450 nm) of the residual stain collected from sample wells). Wells without biofilms added with 200 μL of ruthenium red stain acted as blanks [68].

### 3.5. Effect of Selenium on the Expression of UPEC Biofilm-Associated Genes

The effect of selenium on the transcription of UPEC genes required for biofilm formation and virulence (*fimH*, *fimA*, *sfaS*, *pepG*, and *focA*) was investigated by using real-time quantitative PCR (RT-qPCR) [61,69]. Each UPEC strain was grown separately with or without the SIC of selenium at 37 °C in TSB to mid-log phase in sterile 12-well polystyrene plates. Total RNA was extracted using the RNeasy RNA isolation kit (Qiagen, Valencia, CA, USA). Complementary DNA (cDNA) was synthesized using the iscript cDNA synthesis kit (Biorad, Hercules, CA, USA) and used as the template for RT-qPCR. The amplification product was detected using SYBR Green reagents (Biorad). Relative gene expression was determined by the comparative critical threshold (2^−∆∆*Ct*^) value method using a StepOnePlus^TM^ Real Time PCR system (Applied Biosystems, Carlsbad, CA, USA) and expressed as fold change in expression relative to controls. Data were normalized to the endogenous control (16S rRNA) and the level of candidate gene expression between control samples (not exposed to selenium) and treated samples (exposed to the SIC of selenium) was compared to study the effect of selenium on the expression of each biofilm-associated gene. Gene specific primers were used based on published literature [59], and 16S rRNA gene was used as a housekeeping gene or endogenous control.

### 3.6. Inactivation of Preformed UPEC Biofilms on Urinary Catheters by Selenium

The efficacy of selenium for inactivating fully formed UPEC biofilms on catheters was determined as previously described [20,66]. Latex 12Fr Foley urinary catheters were inoculated with UPEC as described under Section 3.3. Following inoculation, the catheter pieces were sealed and incubated at 37 °C to facilitate UPEC biofilm formation. After 5 days, each catheter piece was washed with sterile PBS to remove unattached cells, sealed at one end, filled with 1 mL of sterile PBS (control) or PBS containing 0.75% (45 mM), 1% (60 mM), and 1.5% (85 mM) of selenium, sealed at the other end, and incubated at 37 °C for 5 days. On days 0, 1, 3, and 5, biofilm-associated UPEC population was determined as described previously. Three catheter pieces were included per treatment at each sampling time and the experiment was repeated three times.

### 3.7. Scanning Electron Microscopy (SEM)

*E. coli* CFT073 biofilm formation and inactivation by selenium on urinary catheters was examined by SEM [70]. UPEC biofilms were formed on catheter pieces as described previously. Following biofilm formation, the inoculated catheter pieces were washed with sterile phosphate-buffered saline (PBS) to dislodge the loosely attached and unattached bacterial cells. Subsequently, they were treated with either 2% (110 mM) selenium or sterile PBS for 1 day at 37 °C. Samples were then fixed in glutaraldehyde-paraformaldehyde-cacodylate buffer (pH 7) at 4 °C for 90 min. Following fixation, catheters were washed with 0.1 M Na cacodylate buffer (pH 7) and post-fixed in 1% osmium tetroxide at 4 °C overnight. The catheters were then rinsed twice for 15 min in distilled water, then dehydrated in serial concentrations of ethanol (30%, 50%, 70%, 95%, 100%, 100% ETOH, 15 min each), and critical point dried (Critical Point Dryer 931GL, Tousimis, Rockville. MD, USA). The dried catheter samples were then mounted on the SEM stub using silver paint and sputter coated with gold/palladium and examined using a scanning electron microscope (Nova NanoSEM 450, FEI, Hillsboro, OR, USA).

### 3.8. Statistical Analysis

The entire study was repeated three times with triplicate samples with a completely randomized design. Data were analyzed using the PROC GLM procedure of SAS (v 9.2, The SAS Institute, Cary, NC, USA) and differences between the means were compared using the least significant difference test. Differences were considered significant when the *p* value was <0.05.

## 4. Conclusions

In conclusion, our study demonstrates that selenium at lower concentrations (SIC) was effective in preventing UPEC biofilm formation on urinary catheters. Further, this inhibitory effect of selenium was found to be associated with a reduction in EPS production and the expression of UPEC genes involved in biofilm formation. Additionally, at high concentrations (MBC), selenium was effective in inactivating preformed UPEC biofilms on urinary catheters. These results suggest that selenium could be potentially used as an antimicrobial catheter lock solution to control UPEC biofilms on indwelling devices. Despite its antibiofilm effect, given the concerns for potential side effects associated with the administration of selenium, additional experiments are needed to determine its safety and to evaluate the efficacy of selenium in comparison with other antibiofilm therapies in vitro and in vivo.
